# Dosimetry-based treatment for Graves’ disease

**DOI:** 10.1097/MNM.0000000000000826

**Published:** 2018-04-02

**Authors:** Steve L. Hyer, Brenda Pratt, Matthew Gray, Sarah Chittenden, Yong Du, Clive L. Harmer, Glenn D. Flux

**Affiliations:** a Department of Endocrinology, Epsom and St Helier University Hospitals NHS Trust; b Department of Physics; c Department of Nuclear Medicine; d Thyroid Unit, Royal Marsden NHS Foundation Trust, Sutton, UK

**Keywords:** benign thyroid disease, dosimetry, Graves’ disease, hyperthyroidism, radioiodine

## Abstract

**Objective:**

The aim of this retrospective study was to assess the long-term outcome of a personalized dosimetry approach in Graves’ disease aiming to render patients euthyroid from a planned thyroid absorbed dose of 60 Gy.

**Patients and methods:**

A total of 284 patients with Graves’ disease were followed prospectively following administration of radioiodine calculated to deliver an absorbed dose of 60 Gy. Patients with cardiac disease were excluded. Outcomes were analysed at yearly intervals for up to 10 years with a median follow-up of 37.5 months.

**Results:**

A single radioiodine administration was sufficient to render a patient either euthyroid or hypothyroid in 175 (62%) patients, the remainder requiring further radioiodine. The median radioactivity required to deliver 60 Gy was 77 MBq. Less than 2% patients required 400–600 MBq, the standard activity administered in many centres. In the cohort receiving a single administration, 38, 32 and 26% were euthyroid on no specific thyroid medication at 3, 5 and 10 years, respectively. Larger thyroid volumes were associated with the need for further therapy. The presence of nodules on ultrasonography did not adversely affect treatment outcome.

**Conclusion:**

A personalized dosimetric approach delayed the long-term onset of hypothyroidism in 26% of patients. This was achieved using much lower administered activities than currently recommended. Future studies will aim to identify those patients who would benefit most from this approach.

## Introduction

The optimal radioiodine treatment strategy for Graves’ disease is controversial as reflected in the differing guidance offered by international guidelines. The American Thyroid Association aims to render patients hypothyroid by administering 370–555 MBq (10–15 mCi) or 5.5 MBq (150 mCi) per gram of thyroid tissue 1. The Royal College of Physicians (UK) recommends a fixed activity of 400–600 MBq for rapid elimination of hyperthyroidism accepting that a significant proportion of patients will require life-long l-thyroxine with this approach 2. In contrast, the European Association of Nuclear Medicine recommends administration of an activity of radioiodine calculated to give an absorbed dose of 150 Gy to the thyroid where the aim is to restore euthyroidism 3.

A fixed activity approach has the advantages of cost-effectiveness, efficiency and early eradication of hyperthyroidism thus avoiding its adverse consequences 4–6. In contrast, an individualized approach on the basis of dosimetry aims to administer the minimum activity of radioiodine necessary to render a patient euthyroid for the maximum length of time in accordance with the ionizing radiation regulations, which are guided by the ALARA (as low as reasonably achievable) principle in radiation protection, that is to keep radiation doses ‘as low as reasonably achievable’ and stipulate that exposures of target volumes must be individually planned 7. The disadvantage of remaining on antithyroid medication for longer with this approach is offset by the delayed need for l-thyroxine replacement since ∼40% of patients on long-term l-thyroxine are either over-treated or under-treated, and hence at risk of associated morbidities 8.

Following an initial study that demonstrated that an absorbed dose of more than 40 Gy iodine-131 (^131^I) was needed to achieve short-term control of hyperthyroidism in Graves’ disease 9, the long-term prospective study presented here was performed to assess the feasibility and effectiveness of an activity administration calculated to deliver 60 Gy to the thyroid. Treatment outcomes, in terms of hyperthyroidism, hypothyroidism and euthyroidism were investigated with up to 10 years follow-up. In addition, clinical and ^131^I kinetic parameters that might predict treatment outcome were examined.

## Patients and methods

### Patients

Over a 10-year period (1999–2009), 284 patients [216 female, 67 male: mean age of 46.2 years (range: 18.1–81.6 years)] referred to the Thyroid Unit at the Royal Marsden Hospital for ^131^I treatment of Graves’ disease participated in this study, which was approved by the Royal Marsden Hospital Research Ethics Committee. Inclusion criteria stipulated clinical and biochemically proven Graves’ disease and no previous radioiodine treatment. All patients provided written informed consent after receiving detailed information about radioiodine treatment including printed radiation protection instructions. Patients with multinodular goitre, autonomous thyroid nodules or cardiac complications (heart failure or atrial fibrillation) were excluded from the study. Patients who were unable or unwilling to come up for the additional six visits required for dosimetry underwent standard treatment with a fixed radioactive iodine dose of 75 MBq.

The diagnosis of Graves’ disease was made according to standard criteria: typical clinical findings and the presence of thyroid-stimulating hormone (TSH) receptor autoantibodies. Those with a diffuse and homogenous pattern on technetium-99m-scintigraphy were classified as typical Graves’ disease (227 patients). The remaining 57 patients with nonuniform uptake on scintigraphy or nodular appearance on thyroid ultrasound scanning were classified as Graves’ disease in a nodular thyroid. Thyroid volume was determined immediately before a tracer administration of radioiodine. Radioiodine kinetics was determined following tracer and therapy administrations of Na^131^I.

The study schedule is shown in Table 1. Patients were advised to follow a low-iodine diet and to stop antithyroid medication commencing 3 days before radioiodine administration and continuing for 9 and 6 days for tracer and therapy, respectively. Tracer and therapy administrations were 14 days apart. This schedule ensured that patients were euthyroid at the time of ^131^I treatment, thus minimizing the risk of thyroid storm. In addition, it allowed tracer and therapy administrations to be given under the same physiological conditions. Those patients with clinical evidence of thyroid eye disease received 0.5 mg/kg oral prednisolone with the radioiodine at therapy and with the tracer administration to prevent ophthalmopathy worsening.

**Table 1:**
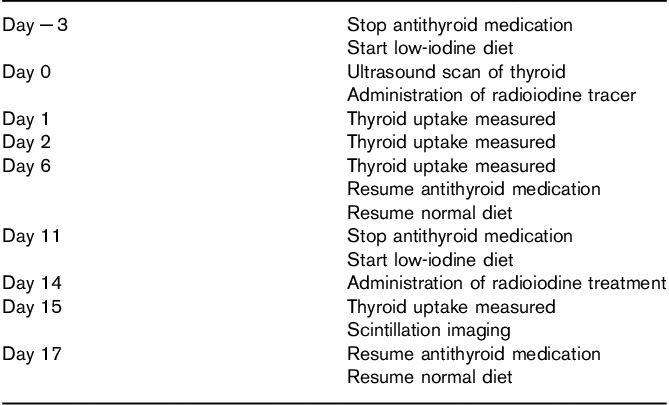
Summary of study schedule

### Measurements

#### Thyroid volume

Thyroid volume was assessed by ultrasound performed immediately before tracer administration. Thyroid volume was calculated from the formula: *V*_lobe_=length (cm)×width (cm)×depth (cm)×*m* (where *m*=0.479) taking the maximal linear dimensions of each lobe on a frozen B-scan image 10. Texture was graded as homogeneous, heterogeneous or nodular.

#### Radioiodine kinetics

Following oral administration of a 0.37 MBq Na^131^I tracer administration, radioiodine uptake in the thyroid was measured using a sodium iodide detector with diverging collimator. Thyroid uptake was determined by comparison of the count rates from the patient’s thyroid and a known activity of ^131^I in a standard neck phantom. Background counts were measured with a lead shield positioned between the front of the collimator and the patient’s neck or phantom. Percentage thyroid uptake was determined at 1, 2 and 6 days following the tracer administration:
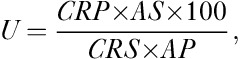
where *U* (%) is the percentage thyroid uptake, CRP is the patient count rate (cpm), AS is the activity of the standard (MBq), CRS is the count rate of the standard (cpm) and AP is the activity in the patient (MBq).

For each patient, the maximum recorded thyroid uptake (*U*) and the uptake at 6 days were used to determine the effective half-life (days). The effective half-life was set to 8 days in cases where it was calculated to be greater than this value.

#### Dosimetry

The activity (*A*) of ^131^I needed to deliver 60 Gy to the thyroid was calculated from the following equation on the basis of the Medical Internal Radiation Dose schema:
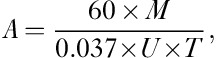
where 60 is the target thyroid absorbed dose (Gy), *M* is the mass of the thyroid (g), *U* is the maximum uptake in the thyroid (%) and *T* is the effective half-life in the thyroid (days).

Radioiodine therapy was administered 1–2 weeks after the tracer study. At 24 h after therapy administration the thyroid uptake was measured using the same system and setup as for the tracer study. Owing to the high ^131^I activity present it was necessary to attach a secondary collimator to the diverging collimator to reduce the sensitivity of the system and thus avoid problems resulting from dead-time. The absorbed dose to the thyroid was then calculated using the thyroid volume and effective half-life calculated in the tracer study.

A gamma camera image was also taken at this time to assess heterogeneity of uptake.

Following radioiodine therapy, antithyroid medications were restarted and continued until patients were clinically and biochemically euthyroid. All patients received radiation protection advice regarding contact with children and time off work in line with standard guidelines. Patients were advised to avoid close contact with young children (<3 years) for 7 days and there was no restriction on work with adults unless contact with pregnant women was possible. Patients attended follow-up at regular visits for a minimum of 12 months up to 10 years with a median follow-up of 37.5 months. At each visit thyroid status was evaluated clinically and biochemically and patients were classified as hypothyroid requiring l-thyroxine (elevated TSH with or without low FT4), hyperthyroid requiring antithyroid treatment (elevated FT4 or FT3 and low TSH) or euthyroid on no specific thyroid medication (normal FT4, FT3 and measurable TSH). The requirement for a further administration of ^131^I was based on clinical assessment. A second administration of ^131^I was considered an outcome endpoint and any such patients left the study to be followed in the standard endocrine clinic.

### Statistical analysis

Statistical analysis was performed using GraphPad Prism and Bell Laboratories statistical computing package (Graphpad software; San Diego, California, USA), ‘R’. Descriptive statistics are provided as mean±SD for normally distributed data or as median with 95% confidence intervals (CIs) and range for non-normally distributed data. Thyroid status was analysed at 6 months, 1, 3, 5 and 10 years. For the purpose of analysis it was assumed that patients who were hypothyroid on l-thyroxine replacement at discharge remained in the same state. For multivariate analysis to compare baseline variables (thyroid volume, age, ^131^I uptake, effective half-life) and thyroid status, we used logistic regression analysis. The coefficients of the independent variables in the regression were transformed to give the odds of a particular treatment outcome. The odds ratios (ORs) are expressed with 95% CIs. The regression was repeated at 3, 5 and 10 years with *P* value less than 0.05 taken as indicating statistical significance in all tests. All *P* values presented are two tailed.

## Results

### Baseline measurements

Baseline characteristics are shown in Tables 2 and 3. In comparison with patients with typical Graves’ disease, those with Graves’ in a nodular goitre were more likely to be male, older and to require more activity to achieve the target 60 Gy absorbed dose. The maximum uptake of ^131^I after tracer and therapy administrations was reduced in nodular glands although the ^131^I effective half-life was slightly but not significantly longer. Only five patients required 400 MBq or more to deliver a target absorbed dose of 60 Gy. The maximum thyroidal ^131^I uptake was very similar for the tracer and therapy (Table 3). The median thyroid volume was greater in the group with nodules. Five patients requiring over 400 MBq had significantly larger thyroid volumes (mean: 56.4 ml; range: 37.9–96.8 ml; *P*<0.05). Median effective half-life in Graves’ and Graves’ with nodules was not found to be statistically significant. Analysis of the whole patient cohort gave a median absorbed thyroid dose of 55.8 Gy (55.03–58.03).

**Table 2:**
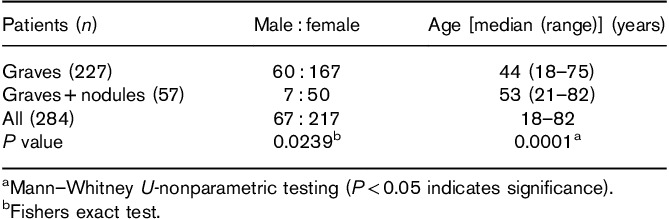
Baseline characteristics with univariate analysis

**Table 3:**
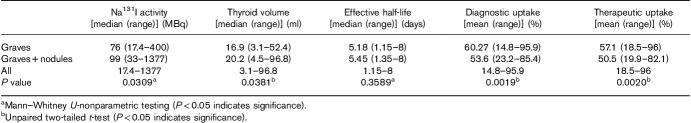
Metrics for calculating a fixed target absorbed dose of 60 Gy to the thyroid

### Treatment outcomes

**Fig. 1:**
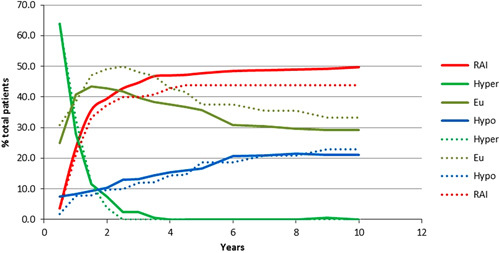
Treatment outcomes for patients with Graves’ disease (*n*=227) (solid lines) and for Graves with nodules (*n*=57) (dotted lines). EU, euthyroidism; hypo, hypothyroidism; hyper, hyperthyroidism; RAI, radioactive iodine.

Clinical outcomes for patients with typical Graves’ disease (*n*=227) and Graves’ in a nodular thyroid (*n*=57) are shown in Fig. 1. At 18 months, 44.2% of all study patients were euthyroid on no specific therapy, 9.0% were on replacement l-thyroxine, 11.6% were still taking antithyroid medication and 35.2% had required a second administration of ^131^I (fixed dose of 75 MBq) because of persistent hyperthyroidism requiring antithyroid medication.

The proportion of patients requiring further radioiodine increased to 43.8% by 3 years but thereafter changed little (47.0% at 5 years, 48.6% at 10 years). No serious adverse effects of radioiodine (post-therapy flare of hyperthyroidism or significant exacerbation of thyroid eye disease) were observed. At 3, 5 and 10 years after administration, 41.4, 37.7 and 30.0%, respectively of patients were euthyroid on no thyroid medication. The proportion of patients becoming hypothyroid increased from 12.9% at 3 years to 17.0% at 5 years and 21.4% at 10 years. After 3 years, a small percentage of patients were still on antithyroid medication usually because further ^131^I therapy was contraindicated. By 5 years no patients remained on antithyroid drugs.

In comparison with patients with typical Graves’ disease, those with Graves’ in a nodular thyroid became nonhyperthyroid sooner and none required antithyroid medication by 3 years (Fig. 1). At 10 years, a slightly greater proportion were euthyroid (33.3 vs. 29.2%) or hypothyroid (22.9 vs. 21.0%) and fewer had required a second administration of ^131^I (43.8 vs. 49.7%).

### Factors affecting treatment outcomes

#### Thyroid volume

From multivariate analysis, increasing thyroid volume was found to be highly significant in the likelihood of a patient responding to a single administration of ^131^I. The ORs (95% CIs) for achieving a nonhyperthyroid state (euthyroidism or hypothyroidism on l-thyroxine) are shown in Table 4. At 3 years, OR was 1.044 (1.014–1.077, *P*=0.005), at 5 years the OR had decreased to 1.04 (1.011–1.074, *P*=0.008) and at 10 years to 1.032 (1.004–1.063, *P*=0.029). Thyroid volume did not correlate with effective ^131^I half-life or maximum percentage ^131^I uptake.

**Table 4:**
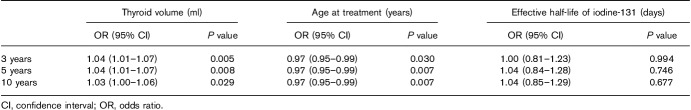
Odds ratios at 3, 5 and 10 years for achieving a euthyroid state

#### Age at treatment

**Fig. 2:**
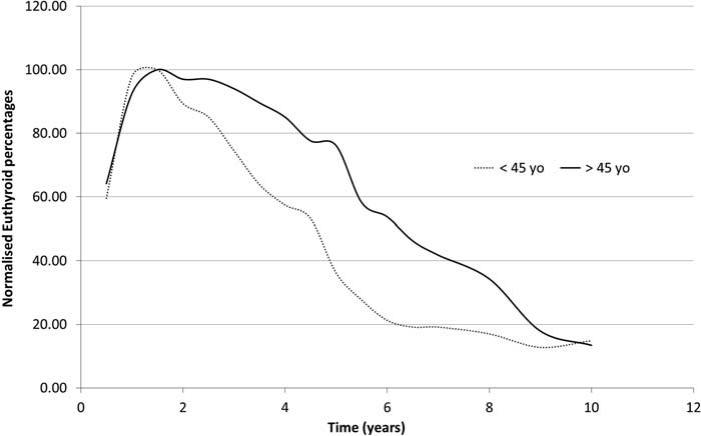
Radiation response to 60 Gy for the euthyroid group on the basis of age at administration.

Increasing age at the point of treatment was significantly associated with the likelihood of a patient successfully responding to a single administration (Fig. 2). The corresponding ORs at 3, 5 and 10 years were very similar at 0.976 (0.955–0.997), 0.97 and 0.969.

#### Effective iodine-131 half-life and maximum iodine-131 uptake

Neither effective half-life nor maximum ^131^I uptake were significantly associated with the need for further ^131^I treatment.

#### Duration of antithyroid treatment

We found no significant association between length of antithyroid treatment before ^131^I treatment and treatment outcomes.

## Discussion

The results of this study indicated that a targeted 60 Gy absorbed dose to the thyroid can delay the onset of hypothyroidism in about a quarter of patients with Graves’ disease. Using the protocol adopted in this study, 62% of patients were treated once with a mean ^131^I activity of 106 MBq. The remainder required a second ^131^I administration usually within 2 years. In patients receiving a single administration, euthyroidism persisted for 10 years and might be expected to continue for an average of 15 years 11. This approach was associated with continued requirement for oral antithyroid therapy in about a quarter of patients for 18 months. It should be noted that none became overtly hyperthyroid. Importantly, we excluded patients with cardiac complications in whom hyperthyroidism needed to be corrected more rapidly.

An absorbed thyroid dose of 60 Gy was chosen for this study after a previous study at this centre demonstrated a high rate of treatment failure at 12 months with lower absorbed doses 9. Howarth *et al*. 12 in a study of patients with Graves’ disease comparing 60 and 90 Gy absorbed doses to the thyroid reported 46% of patients were euthyroid on no specific medication with a median follow-up of 37.5 months. The authors concluded that 60 Gy was a better option than 90 Gy where the aim of treatment was to achieve euthyroidism and to delay the onset of hypothyroidism for as long as possible.

The difference between intended and therapeutically achieved target absorbed doses in Graves’ disease varies widely in previous publications. The coefficient of variation of 22.8% in this study compares well with previous reports of 18–28% 13,14 and is considerably lower than others 15. We noted a significant correlation between pretherapy thyroid volume (and hence thyroid mass) and treatment outcome and this may be relevant when comparing our results with other studies; Thyroid mass (>26 g) has previously been shown to be an important predictor of inadequate response to ^131^I therapy for patients treated with 60–90 Gy 12,13. The relatively small mean thyroid volume (18 ml) in this study is likely to be an important factor in determining the successful outcome of our patients.

Reliance on single measurements of ^131^I uptake at 24 h to determine activity is potentially inaccurate because of the variability of the biological half-life of ^131^I 16,17. Maximum ^131^I uptake occurs after 24 h in 54% of patients with Graves’ disease 18. This may be important in interpretation of the results of previous studies comparing individualized and fixed activity approaches 5,19,20. In our study, ^131^I uptake was measured over 6 days and there was good correlation between diagnostic and therapeutic maximum uptake values. A wide variation in ^131^I effective half-life was noted in this study although the mean of 5.3 days compares well with previous reports 12,13.

A shorter ^131^I effective half-life was found to be associated with an increased risk of hypothyroidism at 3 years in keeping with previous reports 21 although we did not find an association with maximum fractional ^131^I uptake. There may be a need to increase the target absorbed dose in cases where the effective half-life as determined from the tracer administration is found to be short.

Older patients in our study were more likely to become euthyroid after a single administration of ^131^I possibly indicating less severe hyperthyroidism at the outset. Pretreatment with propylthiouracil but not carbimazole may influence treatment outcomes after ^131^I therapy 22. However the use of this drug in our patients was limited to a small number of all ages who developed skin reactions to carbimazole.

The presence of nodules on ultrasonography in Graves’ patients may be an additional confounding factor in the comparison of outcomes from different studies. Patients with autonomous thyroid nodules or toxic adenomas were excluded from our study as with a number of other previously reported studies 12,20. Patients with nodular disease have conventionally been considered to be more radioresistant in comparison to those with typical Graves’ disease 23 although at least one study has noted that these patients may have better long-term results after a single administration of ^131^I therapy 24. Our data suggest that patients with Graves’ in a nodular thyroid have a good response to ^131^I therapy.

There have been few prospective randomized-controlled trials comparing individualized dosimetry versus fixed activity approaches 5,20,25. In an open randomized 12-month trial comparing three fixed ^131^I activities with an individualized absorbed dose equivalent to 70 Gy 20, 50% of patients treated by the individualized dose were euthyroid, 41% still required antithyroid treatment and 9% were hypothyroid. Results for the fixed activity approach were similar. No long-term data was presented. In another randomized trial, 98 patients with Graves’ disease received a fixed activity of 555 MBq and were compared by outcome at 6 months to 107 patients who were administered a planned absorbed dose of 100 Gy 25. A successful outcome, defined as elimination of hyperthyroidism, occurred in 71% of those receiving fixed activity versus 58% in the calculated absorbed dose group. This difference did not quite achieve significance. Long-term outcomes were not reported. Leslie *et al.* 5 randomized 88 patients with Graves’ disease to either (i) 235 MBq; (ii) 350 MBq; (iii) 2.96 MBq/g thyroid (∼60 Gy) or (iv) 4.44 MBq/g thyroid (∼100 Gy). With a mean follow-up of 63 months, the investigators reported no significant differences in clinical outcomes. This study was considerably underpowered and clinically important differences between groups may have been missed.

Irrespective of whether the objective of ^131^I therapy is the elimination of hyperthyroidism or the maintenance of euthyroidism for as long as possible, the principle of ensuring that radiation doses as low as reasonably achievable (ALARA) should be applied 7,26. This principle is not always respected in the treatment of thyroid disease 11. In our study, a very wide range of activity (mean: 106 MBq; range 17.4–1377 MBq) was associated with a targeted absorbed dose of 60 Gy reflecting the poor correlation between administered activity and the absorbed dose. Only five of our patients required the Royal College of Physicians recommended fixed activity of 400 MBq. Even a fixed activity of 370 MBq may result in some two-thirds of patients being unnecessarily over-irradiated 4,11.

It is acknowledged that a dosimetric approach entails more visits to hospital with associated costs and the increased risk of recurrent hyperthyroidism in comparison with a fixed ablative dose. In patients with cardiovascular disease this could have serious consequences, and hence significant cardiac disease is, in our view, a contraindication to this approach. Furthermore, if patients are lost to follow-up, there is the potential for untreated hypothyroidism but this is also the case for patients receiving fixed doses.

The strengths of this study include: (i) a well-characterized cohort of patients all treated in one centre using a standard protocol, in whom cardiac disease was excluded and in whom the presence of thyroid nodules was taken into account in the analysis; (ii) achievement of the targeted absorbed dose of 60 Gy with an acceptable coefficient of variation; (iii) prolonged follow-up with results at 10 years and (iv) outcome measurement of euthyroidism requiring no specific therapy as the preferred measure of successful treatment. A limitation of the study is that we did not directly compare outcomes with patients treated with fixed ^131^I activities in a randomized-controlled trial design.

On the basis of our results, a dosimetric approach would have resulted in nearly two-thirds of patients receiving an average of three to five times less radioiodine compared with standard fixed radiation activities of 370–550 MBq. Further research is needed to identify the optimal absorbed dose for different patient cohorts and investigate which patients would benefit most from this approach.
